# Unexpected Development of Pericardial Effusion in a Patient With Influenza and Secondary Bacterial Infection

**DOI:** 10.7759/cureus.84757

**Published:** 2025-05-24

**Authors:** Pooriya Bahadoran, Daniel Echeverria, Hansani Angammana, Redi Nikollari, Lisa N Glass

**Affiliations:** 1 Medicine, St. Barnabas Hospital Health System, Bronx, USA; 2 Internal Medicine, St. Barnabas Hospital Health System, Bronx, USA; 3 Pulmonary and Critical Care Medicine, St. Barnabas Hospital Health System, Bronx, USA

**Keywords:** alteplase, empyema, fibrinolytic-associated adverse events, intrapleural complications, intrapleural fibrinolytic therapy, pericardial effusion, pericarditis, pleural infection

## Abstract

Influenza poses a significant burden on healthcare systems worldwide, particularly when complicated by bacterial coinfections. Such cases often require intensive medical management. We present the case of a previously healthy young man whose influenza infection was complicated by bacterial coinfection, necessitating complex therapeutic intervention. During treatment with intrapleural fibrinolytic therapy, the patient was found to have developed a large pericardial effusion. While a causal relationship cannot be definitively established, this temporal association raises concern for a potential, previously unreported complication. We present this case to raise clinical awareness of this life-threatening possibility and encourage its consideration in the differential diagnosis when managing similar patients.

## Introduction

Influenza poses a major public health burden globally, with seasonal incidence across all age groups, ranging from 3.0% to 11.3%, and a median of 8.3% (95% confidence interval (CI), 7.3-9.7%) [1]. Annual global estimates suggest between 291,243 and 645,832 respiratory deaths due to seasonal influenza, translating to 4.0 to 8.8 deaths per 100,000 individuals [2]. In the United States, the Influenza Hospitalization Surveillance Network (FluSurv-NET) reported between 114,192 and 624,435 hospitalizations and up to 27,174 deaths per year between 2010 and 2013 [3]. These data emphasize the significant burden that influenza imposes on healthcare systems.

One serious complication of influenza is bacterial coinfection, which can significantly worsen outcomes. During the 2009 influenza pandemic, 30-33% of critically ill patients had a secondary bacterial infection. The most frequently implicated organisms include *Staphylococcus aureus*, *Streptococcus pneumoniae*, *Pseudomonas *species, and *Streptococcus pyogenes* [4,5]. Although coinfections are more common in immunocompromised or chronically ill patients [6], they may also arise in previously healthy individuals.

When pleural infections become loculated or resistant to standard chest tube drainage, intrapleural fibrinolytic therapy may be used. This treatment involves injecting agents such as tissue plasminogen activator (tPA) and DNase into the pleural space to help break down fibrinous septations and facilitate drainage. Although generally effective, this therapy carries a small risk of complications. There are infrequent reports of hemorrhagic pleural effusion following intrapleural fibrinolytic therapy [7]; however, to our knowledge, hemorrhagic pericardial effusion has not been previously described.

We describe the case of a previously healthy young man with influenza and* Streptococcus pyogenes* coinfection who developed a rapidly accumulating, hemorrhagic pericardial effusion following intrapleural fibrinolytic therapy. To our knowledge, this appears to be the first reported case of such a complication occurring in this clinical context. This case highlights a rare but clinically important complication and underscores the need for careful monitoring and consideration of anatomic proximity when managing complex pleural infections with fibrinolytics.

## Case presentation

A 34-year-old male originally from South America presented to the emergency department (ED) with worsening fever, chills, headache, cough, shortness of breath (SOB), and pleuritic chest pain. Two days earlier, he had visited another ED and tested positive for influenza type B. He was discharged with a five-day course of oseltamivir. Due to symptom progression, he returned for re-evaluation.

**Figure 1:**
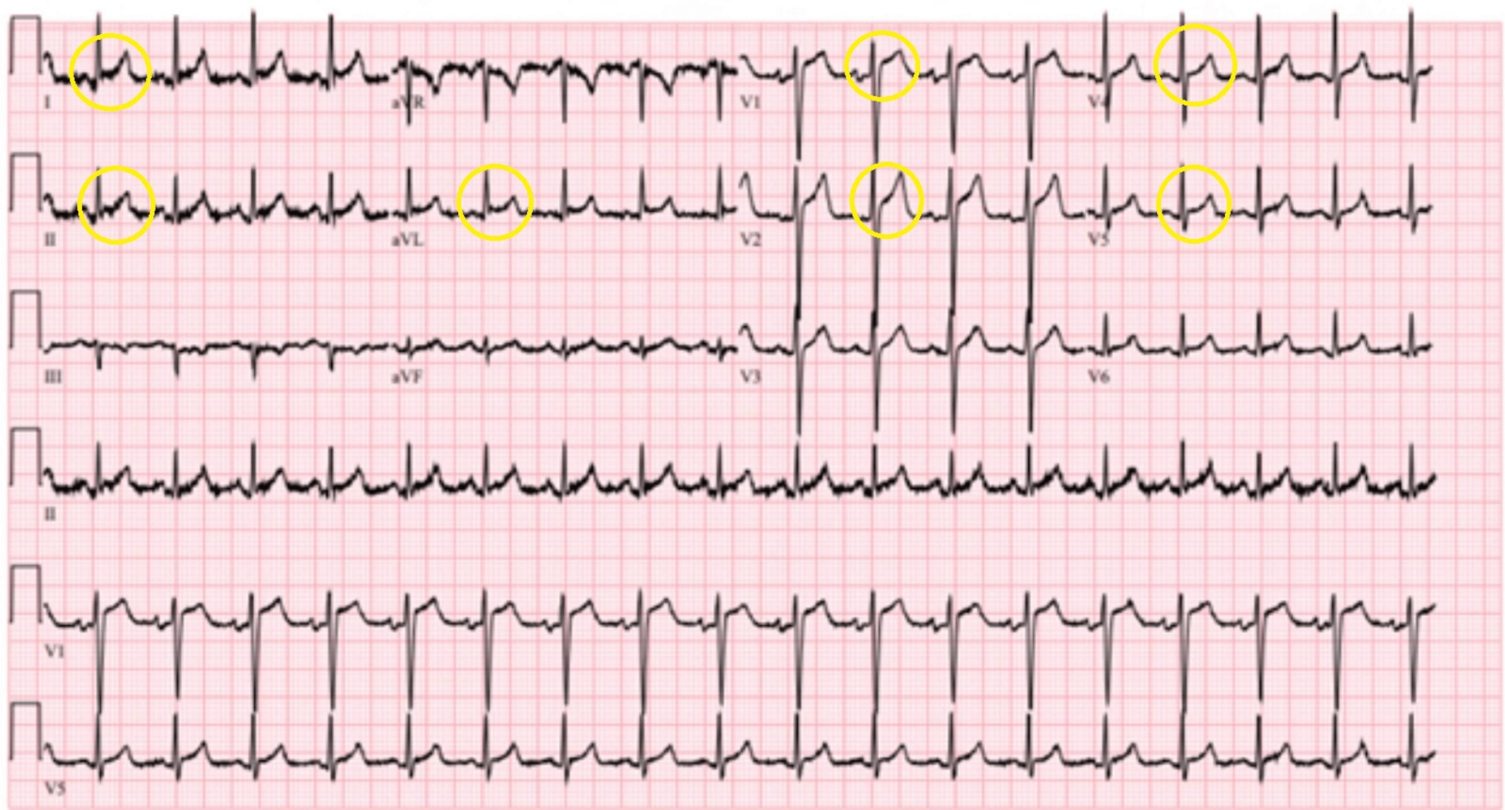
Initial 12-lead electrocardiogram (ECG) on day 1 showing generalized ST-segment elevations. These changes were initially interpreted as early repolarization. In retrospect, they may represent early signs of pericardial involvement, preceding the later development of pericardial effusion. This emphasizes the importance of ECG interpretation in patients with adjacent thoracic infections.

**Figure 2:**
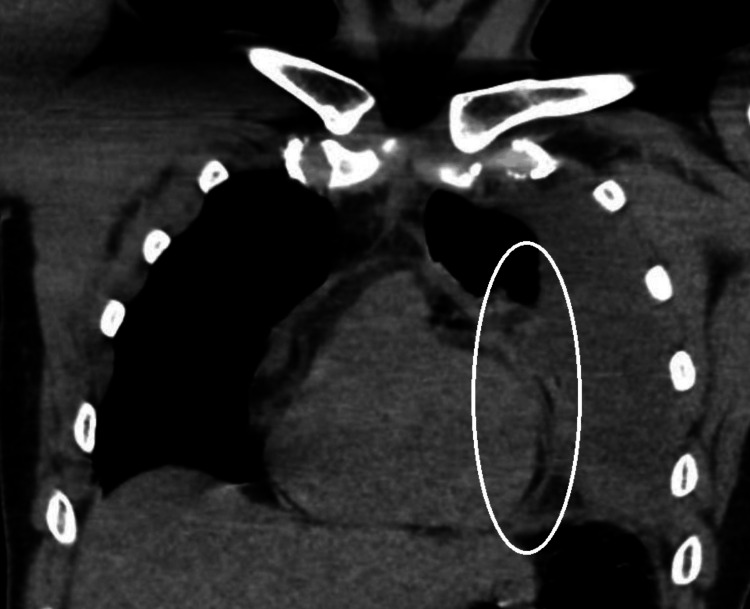
Coronal view of chest computed tomography (CT) on day 1 demonstrating a large loculated left pleural effusion with direct anatomical proximity to the pericardium. This close spatial relationship between the pleural and pericardial compartments may have clinical implications, particularly in the context of intrapleural fibrinolytic therapy. At this time, no pericardial effusion was observed.

On presentation (day 1 of hospitalization), he was noted to have tachycardia, tachypnea, hypoxia requiring supplemental oxygen via nasal cannula, and leukocytosis with a left shift. A repeat influenza test remained positive. Chest X-ray demonstrated a large left pleural effusion, and a 12-lead ECG showed generalized ST elevation, initially interpreted as early repolarization (Figure 1). Chest CT revealed a large, lobulated, and loculated left pleural effusion with associated left lung atelectasis (Figure 2). Notably, the effusion was in direct anatomical proximity to the pericardium.

Blood cultures were obtained, and a left-sided chest tube was placed under ultrasound guidance. Pleural fluid was sent for aerobic and anaerobic cultures. The patient was admitted to the medical floor.

On day 2, he developed progressive drowsiness, increased oxygen requirement necessitating high-flow nasal cannula (HFNC), and worsening tachycardia. Both blood and pleural fluid cultures were positive for *Streptococcus pyogenes* (Group A). Infectious Disease and Critical Care teams were consulted, and he was upgraded to a telemetry-capable unit. Antibiotics were escalated to ceftriaxone 2 g every 12 hours and clindamycin for toxin inhibition. Oseltamivir was continued, and intravenous immunoglobulin (IVIG) was administered for three days.

On day 5 of admission, due to persistent loculated empyema, intrapleural fibrinolytic therapy with tPA and dornase alfa was initiated, administered twice daily for three days (total of six doses). A transthoracic echocardiogram performed earlier that day showed no pericardial effusion, providing a useful baseline.

**Figure 3:**
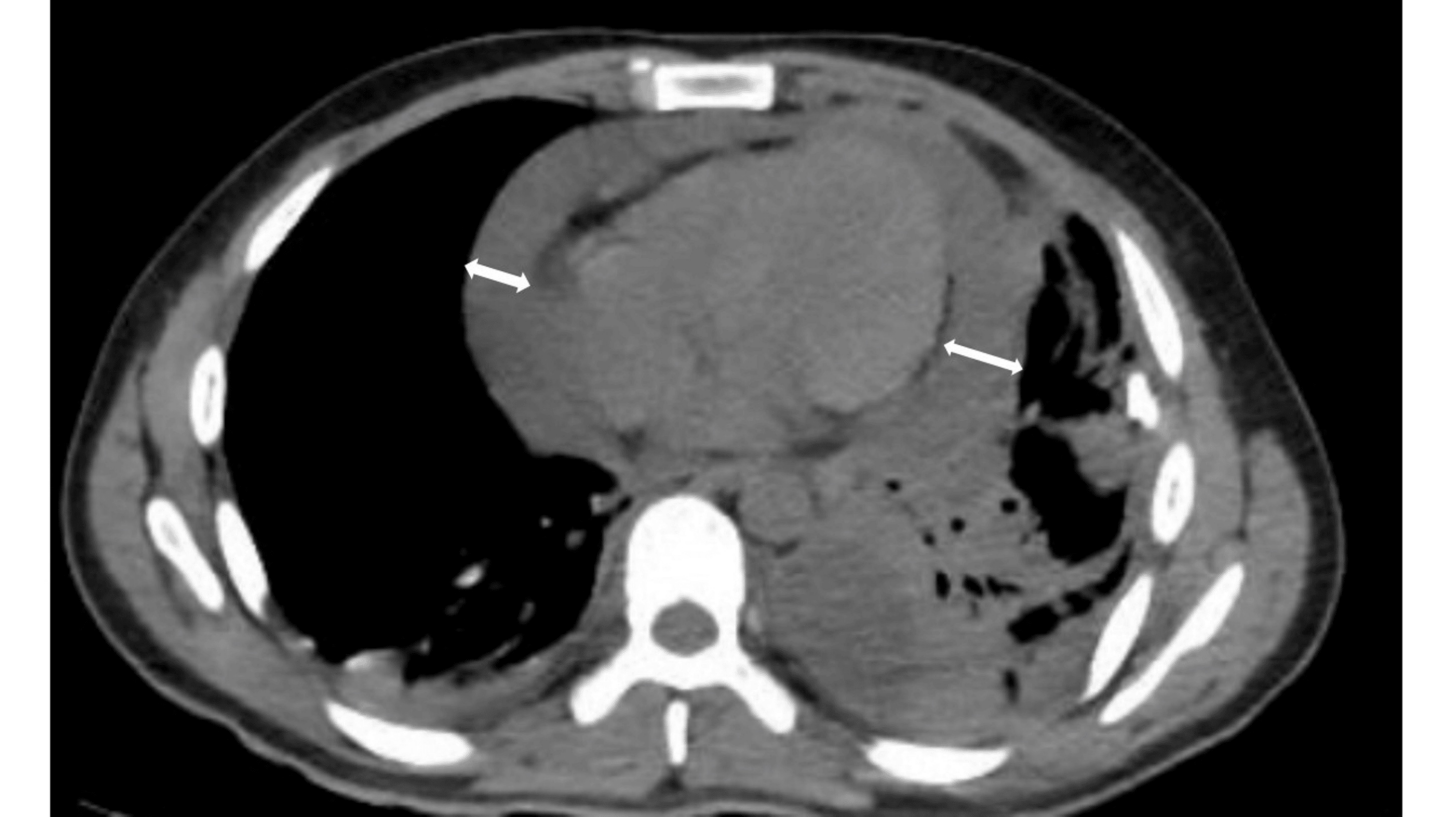
Repeat chest CT on day 10 showing a newly developed, large pericardial effusion. This imaging was obtained after completion of intrapleural fibrinolytic therapy and coincided with a significant drop in hemoglobin and worsening shortness of breath. The pericardial effusion prompted surgical intervention with evacuation of 500 mL of hemorrhagic fluid.

On day 10, the patient remained afebrile but continued to require supplemental oxygen at 2-3 liters per minute via nasal cannula. He had persistent mild tachycardia and shortness of breath with minimal physical activity. A repeat chest CT demonstrated persistent but smaller loculated pleural effusions adjacent to extensive left lower lobe consolidation, as well as a newly developed large pericardial effusion (Figure 3).

Cardiothoracic Surgery (CTS) was consulted, and on day 11, the patient underwent left lung decortication, drainage of multiple lung abscesses, segmental resection of the necrotic left lower lobe, and pericardial window with evacuation of approximately 500 mL of bloody pericardial fluid. Intraoperative findings included dense pleural adhesions, multiloculated pleural and pericardial effusions, thickened pericardium, and multiple lung abscesses.

Cultures from pericardial and pleural fluid and tissue samples were negative except for one pleural tissue culture that grew Staphylococcus warneri, considered a contaminant in the absence of supporting clinical or microbiologic evidence. No additional antibiotics were added.

Postoperatively, the patient showed rapid clinical improvement. His leukocytosis, fever, and tachycardia resolved, and he no longer required supplemental oxygen. He was discharged on amoxicillin-clavulanate 875-125 mg twice daily for four weeks.

Follow-up with Pulmonology and Cardiology was arranged. Given the delayed onset of hemorrhagic pericardial effusion in this case, clinicians should be alert to new or unexplained chest symptoms, worsening anemia, or persistent tachycardia in the days following intrapleural fibrinolytic therapy, particularly when imaging shows anatomical proximity between the pleural effusion and pericardium.

## Discussion

Influenza complicated by bacterial coinfection is more frequently observed in individuals with comorbidities such as advanced age, young children, pregnancy, severe obesity, and chronic underlying medical conditions affecting the lungs, heart, kidneys, liver, nervous system, or immune system [6]. However, our patient was a previously healthy young adult without known risk factors. During the 2009 H1N1 influenza pandemic, *Staphylococcus aureus*, *Streptococcus pneumoniae*, *Pseudomonas *species, and, less commonly, *Streptococcus pyogenes* (Group A *Streptococcus*, GAS) were identified as frequent contributors to bacterial coinfection [4,5]. In this case, both blood and pleural fluid cultures were positive for GAS.

The patient was treated with ceftriaxone, clindamycin, oseltamivir, and intravenous immunoglobulin (IVIG), with clinical improvement. This approach aligns with prior evidence demonstrating reduced mortality in invasive GAS infections treated with clindamycin and IVIG [8].

**Figure 4:**
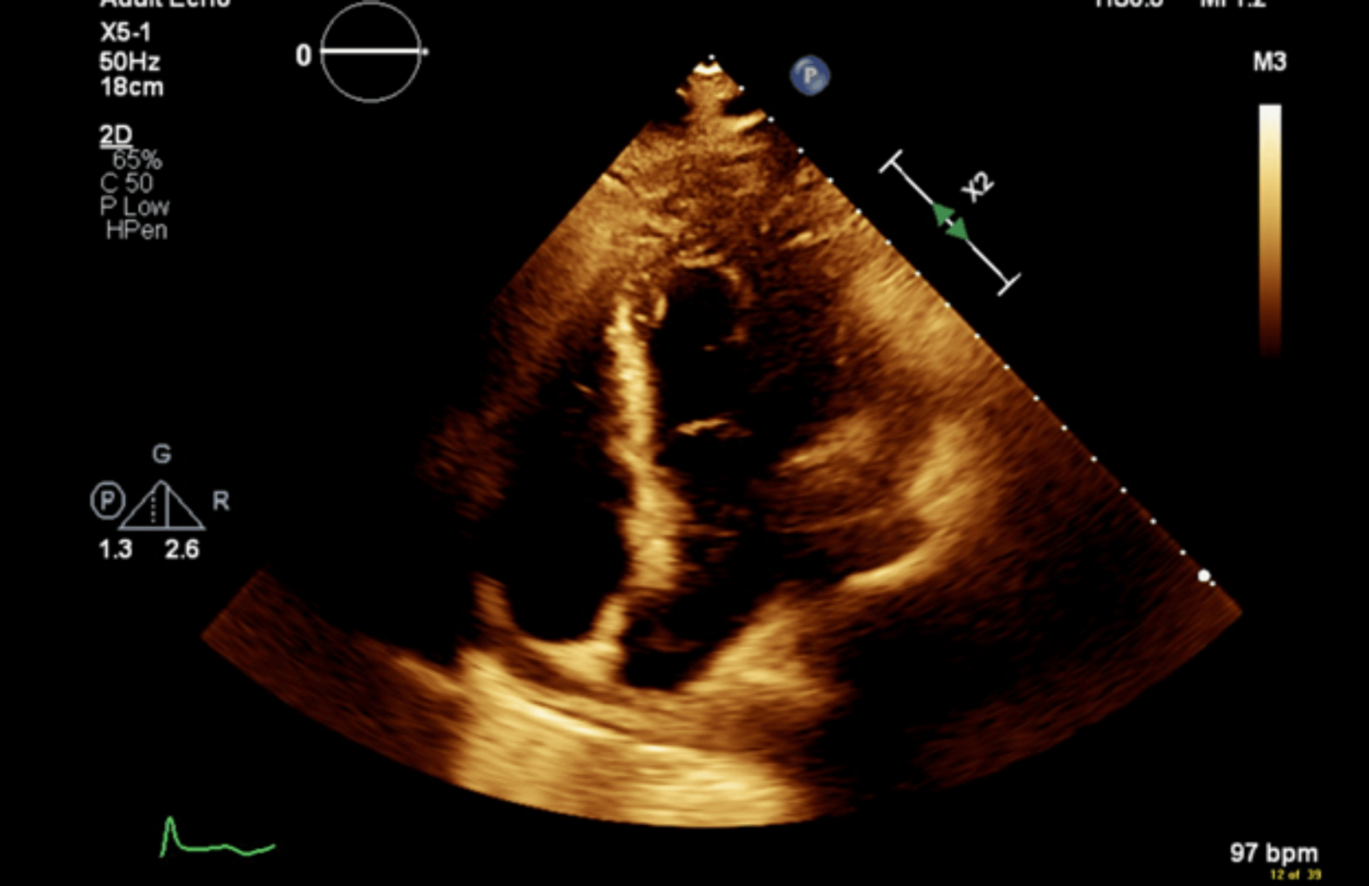
Transthoracic echocardiogram (TTE) performed on day 5 prior to initiation of intrapleural fibrinolytic therapy, showing no pericardial effusion. This study served as a baseline to assess cardiac involvement. The subsequent appearance of pericardial effusion on day 10 reinforces the importance of follow-up imaging in patients receiving intrapleural fibrinolytics near cardiac structures.

Management of complicated parapneumonic effusions typically begins with tube thoracostomy, and if drainage is inadequate, intrapleural fibrinolytic therapy (e.g., tissue plasminogen activator and dornase alfa) is often indicated to promote resolution [9]. In our case, chest CTs obtained on hospital day 1 and day 3 demonstrated no evidence of pericardial effusion. On day 5, a transthoracic echocardiogram (TTE) was performed immediately prior to initiating fibrinolytic therapy, and the absence of pericardial fluid was also confirmed (Figure 4). However, by day 10, a repeat chest CT revealed a new, large pericardial effusion (Figure 3), prompting surgical drainage. On day 11, the patient underwent surgery with a pericardial window, and 500 mL of hemorrhagic fluid was drained.

To better illustrate the patient’s hematologic trajectory and inflammatory response over time, we present the following CBC trend table (Table 1), highlighting key laboratory markers from days 2 to 10. Notably, day 5 represents values immediately before the start of intrapleural fibrinolytic therapy, and day 10 corresponds to the day the large pericardial effusion was first detected on imaging:

**Table 1:** Trend of key hematologic parameters (WBC, hemoglobin, platelets, RBC, and hematocrit) from day 2 to day 10 of hospitalization. Day 5 corresponds to the initiation of intrapleural fibrinolytic therapy, and day 10 coincides with the discovery of pericardial effusion. The table highlights persistent leukocytosis, progressive anemia, and rising platelet counts, which together support the clinical suspicion of hemorrhagic complications. WBC: white blood cell count, RBC: red blood cell count, Hgb: hemoglobin, Hct: hematocrit, pre-fibrinolytics: prior to initiation of intrapleural fibrinolytic therapy, effusion identified: the day pericardial effusion was discovered on imaging

Hospital day	WBC (×10⁹/L)	Hemoglobin (g/dL)	Platelets (×10⁹/L)	RBC (×10⁶/μL)	Hematocrit (%)
Day 2	12.2	12.7	216	4.79	39.3
Day 4	19.4	12.2	292	4.67	38.0
Day 5 (Pre-Fibrinolytics)	19.6	12.7	479	4.85	39.1
Day 7	22.1	10.1	534	3.82	31.4
Day 8	15.7	9.5	578	3.52	29.2
Day 10 (Effusion Identified)	14.7	8.7	693	3.12	26.1

This trend illustrates persistent leukocytosis, progressive anemia, and marked thrombocytosis, with a sharp decline in hemoglobin (from 12.7 to 8.7 g/dL) and hematocrit (from 39.1% to 26.1%) over five days. These changes correlate temporally with the completion of intrapleural fibrinolytics and the emergence of a hemorrhagic pericardial effusion, raising concern for a possible association.

Although hemorrhagic pleural complications following intrapleural fibrinolytics have been reported [7], the development of hemorrhagic pericardial effusion in this context is exceedingly rare. A targeted search of PubMed and Google Scholar using the terms “intrapleural fibrinolytic”, “pericardial effusion”, “bloody pericardial effusion”, and “fibrinolytic complication” yielded no prior reported cases. However, rare complications may be underreported or absent from the indexed literature.

Several plausible mechanisms could explain this complication. One hypothesis is that the fibrinolytic agents may have contributed to local hemorrhage through inflammation-induced vascular permeability, particularly given the close anatomical proximity of the pleural effusion to the pericardium (Figure 2). Alternatively, microperforation or diffusion across inflamed pleuropericardial surfaces could have allowed fluid or agents to track into the pericardial space. Although a direct causal link cannot be definitively confirmed, the temporal association, anatomical context, and a notable hemoglobin drop raise concern for a potential connection.

Infectious pericarditis was also considered. Pericardial cultures were negative after five days; however, the patient was on broad-spectrum antibiotics, which likely reduced the sensitivity of culture-based diagnostics. While PCR or cytology may offer improved diagnostic yield, they were not performed in this case due to rapid clinical recovery following surgical intervention. The absence of fever, resolving leukocytosis, and improving oxygenation at the time of effusion development made active infection less likely.

The patient’s initial 12-lead ECG on day 1 showed generalized ST elevation, initially interpreted as early repolarization. In retrospect, these changes could reflect early pericardial involvement, and we have now included this possibility in the differential diagnosis. Clinicians should be cautious in interpreting such findings in the presence of adjacent thoracic infections or evolving clinical symptoms.

This case highlights a potential but unrecognized complication of intrapleural fibrinolytic therapy in patients with anatomically complex infections. While this therapy remains a cornerstone of empyema management, clinicians should consider baseline and follow-up echocardiography, especially when the pleural effusion abuts the pericardium on imaging. In patients with persistent dyspnea, hemodynamic changes, or new ECG abnormalities, a low threshold for repeat imaging may aid in identifying evolving pericardial complications early.

## Conclusions

Intrapleural fibrinolytic therapy remains a standard and effective approach for managing persistent, loculated pleural effusions. In this case, the patient developed a hemopericardium - a rare and potentially life-threatening complication. Although a definitive causal relationship cannot be established, the temporal association, presence of bloody pericardial fluid, and anatomical proximity of the pleural effusion to the pericardium (as demonstrated on imaging) raise clinical concern.

This case underscores the importance of careful imaging review and consideration of baseline and follow-up echocardiographic monitoring in select patients receiving fibrinolytics, particularly when anatomical relationships may increase risk. In patients whose pleural effusions are in direct contact with the pericardium, clinicians should maintain a high index of suspicion for pericardial involvement, including early signs of pericarditis on ECG or physical exam. If the patient exhibits signs or symptoms of pericarditis from the outset, or if symptoms persist or clinical improvement is less than expected, there should be a low threshold for follow-up imaging to evaluate for newly developed pericardial effusion.

While infection remains a possible etiology, especially in the setting of ongoing antibiotic treatment and negative cultures, the sequence of events in this case suggests a potential association with fibrinolytic therapy. Further investigation and case accumulation are needed to clarify the incidence, mechanisms, and risk factors for this complication.
